# Advancing emergency medical team classification in MENA region: a qualitative study

**DOI:** 10.3389/fpubh.2026.1820087

**Published:** 2026-05-19

**Authors:** Mohamed Abdelaziz, Nikolaos Markou-Pappas, Latifa Arfaoui, Martina Valente, Francesco Barone-Adesi, Luca Ragazzoni

**Affiliations:** 1 IFRC Health Department, Geneva, Switzerland; 2 CRIMEDIM - Center for Research and Training in Disaster Medicine, Humanitarian Aid and Global Health, Università del Piemonte Orientale, Novara, Italy; 3 Eastern Mediterranean Regional Office EMRO, World Health Organization, Cairo, Egypt; 4 Department for Sustainable Development and Ecological Transition, Università del Piemonte Orientale, Vercelli, Italy

**Keywords:** disaster response, Emergency Medical Teams initiative, EMT classification, EMT in LMIC, health system strengthening, MENA region

## Abstract

**Introduction:**

Emergency Medical Teams (EMTs) provide critical surge capacity during health emergencies. While the WHO EMT Strategy 2030 envisions a world in which every country can respond rapidly, effectively, and flexibly to national emergencies, a significant gap persists in the Middle East and North Africa (MENA) region. In this region, low- and middle-income countries (LMICs) lag in the development of classified Emergency Medical Teams (EMTs). The WHO classification system ensures quality-assured medical responses according to the World Health Organization’s classification system. A study was conducted to understand the specific barriers and facilitators of EMT classification in the MENA region to inform strategies for strengthening local and regional preparedness.

**Methods:**

We conducted a qualitative study involving semi-structured interviews with 17 EMTs stakeholders. Participants were purposefully selected from five LMICs in the MENA region (Yemen, Syria, Jordan, Egypt, and Tunisia) and the IFRC regional office, representing a range of roles including EMT process including EMT focal points, project coordinators, and organizational leadership. The data were analyzed using an inductive thematic approach to identify the core themes emerging from the interview transcripts.

**Results:**

Our study identified four principal themes: participants acknowledged the significance of classification in maintaining quality and credibility, however described the process as lengthy, unclear, and challenging. Significant challenges included the perceived rigidity of global standards, particularly in non-clinical logistics and self-sufficiency requirements, the lack of formal training and clear guidance, and inconsistent mentorship. Bureaucracy and inadequate resources were two significant challenges that emerged within the organization. Conversely, robust leadership commitment, specialized technical expertise, and prior deployment experience were regarded as significant enabling factors.

**Conclusion:**

Advancing EMT classification in the MENA region requires a tailored approach that adapts global standards to the local context. Strengthening institutional capacity through training, focused mentorship, and leadership commitment is critical for process completion. To establish a resilient EMT network in LMICs and achieve the EMT strategy, it is imperative to address the challenges and promote the enabling factors.

## 1 Introduction

Disasters triggered by hazardous events that interact with vulnerability and exposure conditions represent a significant disruption to societal structures. These disturbances exceed the capacity of the affected society or community to cope using its own resources (1). Outbreaks, disasters caused by natural hazards, and complex emergencies place a disproportionate burden on low- and lower-middle-income countries (2). These countries often have fragile health systems marked by limited infrastructure, shortages of trained health workers, and inadequate essential supplies. When a disaster strikes, such gaps hinder timely and effective response (3). On top of that, limited disaster preparedness and scarce emergency resources often mean that critical support—like medical care and logistics—cannot reach people in time, amplifying the impacts and making recovery more difficult (4).

Emergency Medical Teams (EMTs) play a vital role in the global health security. They provide additional surge capacity and expertise in response to disasters and health emergencies to the affected countries (5). The EMTs, consist of doctors, nurses, paramedics, and other health experts from all over the world, who can be quickly deployed to treat patients during disease outbreaks, sudden onset calamities, and conflicts, such as the war in Ukraine (6). EMTs are composed of actors from different extractions, such as governmental organizations, civil protection, international humanitarian networks, especially the International Red Cross, and charities/non-governmental organizations (NGOs), such as the Red Crescent Movement, Doctors without Borders (MSF), United Nations, or the for-profit corporate sector (7).

The World Health Organization (WHO) EMT Initiative is a global effort led by the WHO to strengthen national and international surge capacity by supporting, classifying, and coordinating EMTs to ensure timely, high-quality, and accountable medical responses during emergencies. The initiative was established following a review of the 2010 Haiti earthquake response (8), which underscored the need for minimum standards to ensure quality health services in host countries (7). The first publication to classify and set minimum standards for the EMTs was released in 2013 in the context of Typhoon Haiyan in the Philippines (9).

The EMT classification system has evolved, with the WHO having classified 62 teams until December 2025, reflecting the growing recognition of the importance of structured and efficient emergency medical responses. The classification system aims to enhance response effectiveness by ensuring that EMTs meet established criteria for deployment and operations in diverse humanitarian contexts (10).

The IFRC, the world’s largest volunteer-based humanitarian network, has a long history of providing medical assistance in crises, dating back to 1863. In 2021, the IFRC and WHO formalized their collaboration through the “Red Channel Agreement” to implement the Emergency Medical Team (EMT) Initiative. As part of this agreement, the IFRC has aligned its own medical response units with global EMT standards and is now responsible for classifying Red Cross and Red Crescent medical teams using the same processes as the WHO (11).

The EMT 2030 Strategy sets out the strategic directions and priorities for the EMT Initiative at global, regional, and national levels, envisioning “*a world in which every country has the capacity to respond rapidly, effectively and flexibly to national emergencies, leveraging regional and subregional capacities to support vulnerable populations and the people most in need.*” (12) This calls for greater regionalization and a stronger focus on context-appropriate national capacity building.

Considering the increasing complexity of humanitarian crises in the Middle East and North Africa (MENA) region, the need for an integrated disaster response has never been more critical. The diverse nature of disasters, ranging from armed conflicts to natural calamities, demands that EMTs possess not only medical expertise but also cultural competence and an understanding of local sociopolitical dynamics. This is particularly true given that the region is characterized by a high proportion of vulnerable populations, including women and children, who often bear the brunt of humanitarian emergencies (13). The disparities in response capacities among countries in the MENA region demonstrate the need to develop tailored strategies that address the unique challenges faced by low- and middle-income countries (LMICs). Natural disasters deliver a double blow to the already fragile healthcare systems of LMICs in the MENA region, straining them both during the crisis and long after. The repercussions manifest as a network of increasing disease outbreaks, deteriorating chronic diseases, increased mortality and morbidity, and the breakdown of essential infrastructure (14).

Until 2025, the MENA region had only one classified EMT, the Saudi Disaster Response Team, with no recognized teams from LMICs. The lack of classified EMTs in LMICs underscores a significant gap in emergency preparedness and response capabilities. The urgent need for capacity building in these countries is further amplified by the region’s unique socio-political landscape, which often complicates the delivery of aid and medical assistance across border (15).

Nevertheless, the persistent challenge of EMTs classification in LMICs across the MENA region highlights a gap in understanding the mechanisms hindering the advancement of the localization agenda.

To support the practical implementation of EMT localization, this study aims to identify barriers to the formation and accreditation of EMTs in LMICs within the MENA region. It examines challenges in meeting minimum standards and classification requirements, as well as factors influencing the classification process. Specifically, the study aims to: (1) explore stakeholders’ perceptions of the EMT classification process and its standards; (2) identify barriers to meeting EMT classification requirements; and (3) identify enabling factors and propose recommendations to strengthen EMT classification efforts.

## 2 Methods

### 2.1 Study design and sampling strategy

A qualitative study design was employed. Data collection involved semi-structured interviews with key stakeholders, using the EMT framework.

A purposeful sampling strategy was employed to select participants with relevant experience and knowledge of EMT in LMICs in the MENA region. The participants included healthcare professionals, EMT coordinators, and representatives from organizations that expressed interest in participating in the WHO EMT classification through either the WHO EMT or the Red Channel Agreement. The sample size was not predetermined; recruitment continued until data saturation was achieved. The final sample was considered adequate for this qualitative study, consistent with established guidance indicating that purposive sampling is appropriate for achieving depth and thematic saturation in qualitative health research.

Participants were recruited through an email invitation sent to all candidate Emergency Medical Teams (EMTs) in low- and middle-income countries (LMICs) in the MENA region. The invitation outlined the purpose of the study, its objectives, and the importance of their contribution. Those interested were requested to reply to the email to indicate their willingness to participate, and only those who provided informed consent were included in the study. Inclusion criteria were: (1) currently working on an EMT classification project or having directly participated in one recently; (2) affiliated with a candidate EMT organization in an LMIC in the MENA region, or with organizations responsible for classification (WHO or IFRC); and (3) willing to provide informed consent. Individuals with no direct involvement in EMT classification activities were excluded.

### 2.2 Study setting

This study was conducted within the WHO and IFRC Middle East and North Africa (MENA) regional frameworks, specifically targeting Low- and Middle-Income Countries (LMICs). The scope is delimited to national entities currently engaged in the Emergency Medical Team (EMT) Global Classification process. This includes teams at various stages of the lifecycle—from the formal submission of an Expression of Interest (EOI) to those undergoing active mentorship and self-assessment. By focusing on this “classification pipeline,” the study captures the transitional dynamics of professionalizing disaster response within resource-constrained and high-volatility environments.

### 2.3 Data collection

The interview guide was developed based on a comprehensive literature review, reviewed by two Arabic-speaking qualitative research experts (MA, LA), and subsequently piloted with two EMT team members from different regions (Europe and Asia) to ensure clarity, relevance, and alignment with the study objectives. Semi-structured interviews were conducted with participants who expressed interest in the study and met the inclusion criteria for the study. The interviews explored key themes, including challenges in meeting the WHO EMT classification standards, obstacles encountered during the classification process, and factors influencing the classification and operational processes of EMTs (Supplementary file 1).

Interviews were conducted via video conferencing tools or face-to-face, depending on the participants’ preferences and availability.

### 2.4 Data analysis

This study employed inductive thematic analysis to systematically identify and categorize patterns within the qualitative data gathered from the semi-structured interviews. Inductive thematic analysis allows themes to emerge directly from the data instead of forcing existing categories, which improves our understanding of the challenges EMTs face in LMICs in the MENA Region. This approach aligns with the principles outlined by Braun and Clarke (16), who emphasized the importance of developing codes and themes based on data to capture the nuanced experiences of participants. By applying inductive coding, the study effectively highlighted the unique obstacles and factors influencing the classification and operational processes of EMTs, providing a rich, contextualized understanding of their challenges in meeting WHO standards.

Interviews were conducted in Arabic, audio-recorded (with participant consent), and transcribed for further analysis. The interviews conducted during the study were recorded to ensure that all participant responses were captured accurately for further analysis. After the interviews were completed, the transcripts were translated into English. Translation accuracy was ensured through a back-translation process: a bilingual researcher independently translated the English transcripts back into Arabic, and any discrepancies were resolved through discussion until consensus was reached.

To ensure the reliability and validity of the findings, two researchers independently reviewed the interview transcripts. The initial phase of the analysis involved developing primary codes that served as foundational categories to organize the data. Then collaborative effort was done to reconcile any differences in the initial coding and create a cohesive set of codes that accurately reflected the data collected. The final coding framework was developed to be comprehensive and representative of the participants’ voices. We utilized ATLAS.ti, a qualitative data analysis software, to code our data. Data saturation was reached by the 17th interview, with no new themes emerging after the 14th, confirming sample adequacy.

The credibility and dependability of the data were enhanced by utilizing a single, qualified researcher to conduct all the interviews consistently. The thematic analysis of the participants’ statements supports the transferability of the study’s conclusions to other contexts, ensuring their relevance and congruence with the participants’ perspectives.

Confirmability was ensured through a documented audit trail of analytical decisions, codebook development, and memos. Trustworthiness was further enhanced through member checking, with a subset of participants validating the findings.

Regarding researcher reflexivity, the lead researcher is a public health professional with direct experience in humanitarian response and familiarity with the EMT initiative. While this facilitated rapport with participants, it also necessitated deliberate reflexive practices to minimize potential bias. To address this, a reflective journal was maintained throughout data collection and analysis, and emerging interpretations were regularly discussed and cross-checked with co-investigators. These measures were implemented to reduce the influence of prior assumptions and ensure that findings remained grounded in participants’ perspectives.

### 2.5 Ethical considerations

Ethical approval was obtained from Comitato Etico Territoriale Interaziendale AOU Maggiore della Carità di Novara (Approval No. 24-6629, dated 21 March 2023). Informed consent has been secured from all participants, ensuring their understanding of the study’s purpose and their right to withdraw at any time. Confidentiality and anonymity of participants was maintained throughout the research process. Only the lead investigator and the co-investigators had access to the recordings and anonymous transcripts.

## 3 Results

A total of 17 participants were interviewed, representing EMTs from five LMICs in the MENA region and the IFRC regional office. Participants were distributed as follows: Yemen (*n* = 2), Egypt (*n* = 5), Jordan (*n* = 3), Syria (*n* = 2), Tunisia (*n* = 2), and organizations responsible for classification (*n* = 3). As shown in Table 1, the participants had an average of 10.4 years of humanitarian experience. Roles during the classification projects included EMT focal points (*n* = 5), project coordinators (*n* = 3), heads of departments within the applying organizations (*n* = 3), technical advisors (*n* = 3), and regional coordinators (*n* = 3).

**Table 1:** Demographic profile of interviewees.

Participant code	Education background	Role	Years in humanitarian experience
1	Public health	Project coordinator	5
2	Industrial engineering	Head of logistics	20
3	Public health	Senior officer	3
4	Public health	Project coordinator	10
5	Community medicine	Project coordinator	15
6	International collaboration	Organization CEO	15
7	Public health	Regional head of health	10
8	Emergency medicine	Head of emergency health authority	6
9	Humanitarian WASH	WASH coordinator	7
10	Hospital management	Head of health department	15
11	Engineering and disaster management	Head of logistic	25
12	Nursing	Regional Senior officer of emergency health	10
13	Clinical psychiatry	Senior officer for mental health	8
14	Medicine	Deputy manager of EMT	1
15	Logistic	Operations manager	4
16	Public health	Emergency heath coordinator	14
17	Public health	Regional emergency health coordinator	9

A thorough study of the interview data identified four primary themes, each containing multiple interconnected subthemes. The four themes jointly reflect the participants’ experiences and perspectives concerning the Emergency Medical Team (EMT) classification procedure and its minimal standards. The first theme explores participants’ understanding, interpretations of the existing classification process’s objective, and experiences with its implementation. The second theme examines challenges inherent in classification, encompassing obstacles relating to adherence to technical standards and the navigation of procedural requirements within the classification process. The third theme addresses the facilitating factors that enabled or encouraged the teams’ progression through the classification process. The fourth theme articulates participants’ suggestions for enhancing standards, the classification process, and the organizational preparedness of teams seeking to obtain classification status.

### 3.1 Perception of the current process

#### 3.1.1 Importance of the classification

Participants found that meeting the minimum standards is a fundamental step toward ensuring that the care provided is safe, consistent, and high quality. Participants concluded that adhering to minimal standards, teams can respond in a coordinated and professional manner which enhancing the confidence and satisfaction of the affected populations and improving operational efficiency during deployments. Moreover, participants felt that meeting these standards played a key role in building the credibility of EMTs for both affected communities and national and international partners:

*“If EMT did not have standards, every entity would handle it differently, which is detrimental in this case where people’s lives are at stake.” P1.*

Participants stressed that EMT classification strengthens local health systems and capitalizes on national resources for effective emergency response. They observed that classification facilitates regional deployment by validating a team’s technical and operational preparedness, while a common language, cultural comprehension, and contextual awareness improve acceptability and efficacy. Moreover, participants regarded classification as a vital mechanism for resource mobilization, since it conveys credibility to donors, partners, and governmental entities. They acknowledged that classified EMTs enhance global health security by integrating into a coordinated, quality-assured EMT network capable at addressing cross-border emergencies.

“*Classification in general makes us distinguished. When it comes to EMT, it is a completely different realm because of humanitarian aspects. When we have a classified EMT, we’ll be able to respond to any disastrous situation in the region, especially that our Middle East region has been constantly suffering from conflicts for quite long now.”* P14.

#### 3.1.2 Experience of the process

Most participants (17 of 18) described their experiences with the EMT classification process as challenging and disappointing. They described it as a lengthy, difficult to navigate, and lacking clarity process. Participants described the steps and expectations of each stage of the classification process as ambiguous.

*“It’s not easy. This is lengthy and complicated process. We need to go with people who are looking for this accreditation step by step to walk with them through the process because it is not clear and has multiple sectors. So, we need to consider a more pragmatic approach sometimes” P7.*

Participants frequently remarked that the process appeared to be designed with high-resource settings in mind, making it less accessible or practical for teams operating in more resource-constrained settings. The absence of tailored support or context- specific guidance to assist participants through the entire process was cited by several as the reason for their frustration, confusion, and disappointment. In contrast, only one participant described their journey toward classification as smooth and straightforward, attributing it to their prior knowledge of the EMT initiative and strong technical experience.

Most of the participants (15 out of 18) reported receiving no training on EMT standards, while the remaining three received some forms of training have received some sort of training.

*“Not up till now. Neither myself nor anyone inside the Red Crescent has ever received any training courses on standardization” P5.*

### 3.2 Challenges in meeting the minimal standards and classification

#### 3.2.1 Challenges with the standards contents

Participants identified several key challenges in implementing the minimum EMT standards in their respective settings. One of the most frequently mentioned challenges was the perceived inflexibility of the standards. They noted that many standards assumed high-resource settings not considering the limitations of local contexts, such as infrastructure constrains, workforce shortages or different care delivery models.

Standards contextualization was seen as essential to ensure technical appropriateness, cultural relevance, and operational feasibility. Moreover, participants highlighted the varying interpretations of the standards among different stakeholders, which sometimes led to confusion, miscommunication, and inconsistent implementation.

*“Standards are not the Quran or the Bible. I have dealt with standards throughout my work history. And I used to face situations while we were working and providing services on the ground, we came across standards that were practically inapplicable.” P5.*

Nonclinical standards were particularly found to be challenging in implementing during EMT classification. The standards related to support service WASH and logistics founds to pose significant operational challenges. Specifically, the requirements for full self-sufficiency in food, water, power supply, and waste management were considered difficult to achieve in resource-limited or unstable contexts. The requirement to provide reliable transportation was frequently cited as a challenge by the participants. Moreover, participants highlighted that establishing and maintaining these nonclinical systems demanded specialized expertise, technical support, and financial resources, which were not always readily available or easy to mobilize.

*“In Gaza’s situation, for example, we can build a field hospital and provide a 14-day safety stock. But after the 14-day period is over, we might fail to get any shipments into Gaza.” P15.*

Clinical standards are generally described as straightforward to implement, although areas such as mental health and palliative care cited as more challenging due to the need for specialized expertise, resources, and cultural sensitivity.

*“The clinical one is straightforward; it is not difficult to adhere to because it is clinical practice. Even in any country, you’ll see most of it is straightforward.” P7.*

#### 3.2.2 Challenges with the classification process

Participants frequently reported a lack of awareness regarding the stepwise EMT classification process. For many teams, the process felt complex and poorly communicated, with limited clarity on what was expected at each stage and how to prepare best. This lack of structured guidance contributed to confusion and uncertainty, particularly among teams implementing the process for the first time or in settings with limited prior exposure to the EMT initiative.

Participants also expressed concerns regarding their limited understanding of the integration of the EMT classification into the broader national emergency health response frameworks, which has undermined the support and resources allocation. In several cases, EMT classification was not prioritized at the institutional or governmental levels.

Another key concern that was reported is the lack of access to precise technical guidance. Participants often highlighted the shortcomings of mentor engagement, noting insufficient in-depth help, minimal practical guidance, and insufficient supportive feedback from the mentors. This left teams unclear regarding the necessary adjustments along with whether they were advancing in the appropriate direction.

Communication gaps were ongoing issues. Participants viewed communication with organizations responsible for classification (WHO/IFRC) as inconsistent, ambiguous, or unresponsive, leading to delays and reducing motivation. The lack of transparency regarding timelines and the absence of a clear project plan were reported to have led to frustration among team members.

Collectively, these factors were perceived as the main obstacles in the classification process. Participants emphasized that addressing these gaps, particularly through clearer communication, structured guidance, and stronger mentorship, is essential for making the EMT classification process more accessible, efficient and equitable.

*“So, the challenge we are facing now is we do not have a monitor, direction, and training on how to reach within this time, because there is no specific timeframe” P6.*

#### 3.2.3 Internal challenges

Participants highlighted several internal organizational barriers that complicated the classification process. A major concern was internal bureaucracy, which often slowed decision-making and created administrative bottlenecks that hindered project progress. Lengthy internal procedures and complex approval chains delayed critical actions and led to a loss of momentum.

Financial limitations were frequently identified as a significant challenge; specifically, constrained budgets hindered the obtaining of essential equipment, the upgrading of facilities, and the recruitment of requisite technical expertise.

The insufficient levels of dedicated human resources were identified as a critical concern, since staff allocated to the EMT classification process frequently managed multiple responsibilities or had restricted availability, hindering their ability to retain focus and provide consistent leadership. Participants emphasized that establishing a dedicated core team that focuses only on the classification effort would significantly enhance coordination, continuity, and accountability in the process.

Leadership commitment was described as inconsistent or fragile. Competing priorities, such as responding to ongoing emergencies or meeting donor requirements, often draw attention and resources away from classification efforts. This change in focus hindered the upholding of organizational alignment and support during the extended course of the entire process.

Participants acknowledged that, in addition to structural and resource-related challenges, profound cultural and capacity-related obstacles are hindering the process. In several organizations, there was a lack of understanding regarding international quality standards or a deficiency of internal champions to drive the transformation. Inadequate technical knowledge, particularly in logistics and Standard Operating Procedure development, slowed the team’s progress. The lack or inadequacy of specialized training programs led workers, despite their motivation, to be devoid of the necessary resources and frameworks for achieving the required standards.

*“This process needs stable personnel until implementation is all over, and then they can train other teams on using the required equipment.” P1.*

### 3.3 Facilitating factors

Participants also acknowledged several organizational strengths and capacities that could significantly facilitate the EMT classification process. Chief among these was the presence of strong commitment of the leadership, which not only valued the classification but also actively promoted it as essential to support the team and system building. In addition, the timely recruitment of technical experts with relevant field experience was considered essential, bringing in the kind of practical knowledge and focused expertise needed to navigate the process effectively.

*“The most important factor is commitment of leadership.” P10.*

Participants also emphasized the importance of framing the classification as a long-term growth opportunity and a strategic investment in quality, accountability, and readiness, rather than merely a bureaucratic process. This mindset shift was seen as pivotal in generating internal buy-in and sustaining momentum throughout the process.

Other enabling factors included regular training initiatives and awareness-raising sessions that helped build institutional knowledge and engage team members at different levels. The presence of a well-established and highly responsive supply chain also played a critical role in ensuring that logistical and operational needs could be met efficiently. Moreover, experience in previous emergency deployments is considered a valuable asset. Securing sufficient financial resources early on was described as fundamental to building the necessary infrastructure and ensuring the overall sustainability of the classification process.

Participants identified several key forms of support that organizations responsible for classification could offer to help ease and guide the EMT classification process, including assistance in developing a clear and realistic action plan, complete with well-defined timelines and milestones.

In addition, structured training programs tailored to different roles within the team were considered crucial, as they would help ensure that all team members had a shared understanding of the classification requirements and felt confident in their contributions.

Finally, ongoing technical support and close mentorship from organizations responsible for classification throughout the process were highlighted as being particularly valuable. Participants emphasized that having someone to turn to for clarification, encouragement, or troubleshooting at each stage could significantly help sustain momentum and effectively navigate challenges.

*“There should be an information bank for the standards, where countries with previous experience put their standards as a know-how showoff, especially that information in the humanitarian field is not a military secret anyway; it’s a field where all countries need to cooperate.” P2.*

### 3.4 Recommendations

#### 3.4.1 Recommendation for standards

When asked about their recommendations for improving the standards, the participants suggested several key areas for improvement: They emphasized the need for standards to be more context-specific and adaptable to different operational scenarios, tailored to reflect the diverse realities that teams face in different countries, health systems, and emergency settings. Greater flexibility in self-sufficiency requirements was also recommended. Additionally, participants proposed the development of a standards ‘explanation bank’ to break down complex requirements into practical, actionable steps, along with the provision of more technical resources to support implementation:

“*We need to break down or have more explanation of what this means, the content of these standards—or sub-content; let us say—to make it clear for those entities applying for this” P7.*

Flexibility was another important point raised, particularly in relation to self-sufficiency. While the principle of self-sufficiency is widely acknowledged as critical for EMT deployment, several participants noted that a more graduated or scalable approach would be helpful. This could allow teams at different stages of development or with varying resource capacities to work toward full compliance realistically, pragmatically, and in a phased manner, rather than being held to a rigid standard.

Participants expressed a strong desire for a centralized information repository containing real-world examples of how other countries have interpreted and applied classification standards and offered clear, practical guidance for implementation. Such a resource could serve as a practical toolkit, showing different options for meeting minimal standards, sharing lessons learned from other teams, and breaking down complex or technical requirements into accessible, step-by-step instructions. One concrete suggestion was the creation of a standard “explanation bank,” complete with examples, user cases, and adaptable templates, to improve understanding of standards interpretation and foster greater confidence in implementation.

#### 3.4.2 Recommendation for the process

When asked for recommendations to improve the classification process, the participants provided several thoughtful and actionable suggestions. One of the most frequently (11 out of 18) mentioned ideas was the importance of starting the process with a team-wide orientation workshop to clarify the steps, roles, and expectations of the process. Such workshops would facilitate building a shared understanding of the classification process, clarify expectations, and align everyone around common goals.

Participants also emphasized that the process should begin with a clear explanation of the purpose of classification and how it fits into the organization’s broader objectives and strategies. Aligning the process with strategic objectives would help teams see it not as an external ask but as an investment in quality, accountability, and long-term preparedness.

Participants recommended breaking the process down into smaller, clearly defined tasks to avoid the team getting overwhelmed and to make progress more manageable.

*“When an organization comes to look at the whole process at once, you feel frustrated. So, I think it’s very important that we not only divide the process itself, that we not only divide it into bite sizes.” P3.*

This step-by-step approach would allow for focused efforts, easier progress tracking, and reduced pressure on teams that are already balancing multiple responsibilities. In addition, participants stressed the importance of clearly explaining the role of the mentor from the beginning, including who they are, what kind of support they will offer, and how they will engage with the team throughout the process. Clarifying this early was seen as key to building trust and maximizing the benefits of the relationship.

Several participants also highlighted that mentoring should not follow a “one-size-fits-all” approach to be effective. It should be culturally sensitive and responsive to the local context, including language preferences, organizational dynamics, and decision-making styles. Having a mentor who understands and respects the local environment is seen as the most effective approach for guiding teams through complex processes. In addition, the importance of having timely and constructive feedback at each stage was noted by the participants as a key factor in avoiding delays and frustrations.

Participants also felt that gaining buy-in from leadership was vital. Raising awareness among decision-makers about the value of classification was seen as critical for mobilizing resources, prioritizing actions, and maintaining institutional commitment.

Fostering peer support to create more opportunities for experience sharing and learning from other EMTs who had already undergone the classification process was strongly encouraged.

#### 3.4.3 Recommendation for organizations

Participants offered several recommendations for organizations considering applying for EMT classification, drawing on their experiences and lessons learned throughout the process. The importance of ensuring that the classification effort is fully aligned with the organization’s strategic priorities and long-term vision was emphasized. Internal alignment across departments and units was also highlighted as a crucial success factor. Participants noted that the EMT classification process is the responsibility of multiple sectors and department teams, with many cross-cutting areas of the same organization requiring active coordination between the medical, operational, administrative, and technical divisions. Alignment among departments and the establishment of proper communication lines would facilitate faster decision-making, reduce delays and confusion, and provide more coherence among the team.

*“The organization needs to have a real desire to excel in this type of activity. Secondly, it can be made in-house or outsourced because not everything has to be done in-house” P2.*

Another key recommendation is to invest in building and retaining skilled workforces. Participants stressed that recruiting, retaining, and continuously training qualified staff are critical for building the technical capacity required to meet the classification requirements. Building and maintaining a team with practical field experience throughout the process helps ensure continuity, sustain institutional memory, and support its long-term sustainability.

Participants also advised conducting a thorough cost analysis at the beginning of the project and developing a robust financial forecast to ensure that adequate resources are available throughout the classification journey.

Strong and visible leadership commitment was identified as a key enabler in all phases. Participants recommended modularizing the EMT setup wherever possible. By structuring teams in a modular way, where components can be activated, scaled, or adapted based on the type of emergency or deployment need, organizations can improve their flexibility and readiness.

Furthermore, strong capacity in logistics, supply chain management, finance, and administration was considered as a must for ensuring operational resilience, and compliance with the technical requirements of the classification.

*“The most important thing to improve the process is to have team members unchanged throughout the process. This will help ideas remain coherent and linked together” P14.*

## 4 Discussion

This study aimed to explore the challenges and barriers faced by LMICs in the MENA region in pursuing EMT classification. The delivery of comprehensive, accessible, and high-quality health services during emergencies depends heavily on pre-established, rapidly deployable, and quality-assured EMT and surge response capacities (16). Developing the resilience of health systems is essential to ensure that they can withstand sudden shocks and ongoing chronic pressures. This need is particularly important in LMICs, where limited resources and financial constraints often hinder health surge response capacity (17).

The findings of this study highlight the complex and multilayered nature of the challenges in EMT classification in such settings. These challenges span a range of issues, including gaps in understanding and applying classification standards, procedural and bureaucratic hurdles in the classification process, and internal and external organizational constraints that affect the readiness and sustainability of EMTs.

These results are consistent with a growing body of evidence emphasizing the critical importance of standardization and accreditation in the delivery of medical services. They are fundamental tools for safeguarding quality, consistency, and safety of care across various settings. Standardized clinical procedures to ensure that patients receive care that meets established minimum quality and safety standards, regardless of context (18).

As participants in this study stressed, classification efforts extend beyond technical standards; they enhance credibility, support health system strengthening, enable coordinated, humane, and effective care, and enable interoperability with other actors during crises. These practices are about saving lives, maintaining dignity, and providing reliable, compassionate care, not just systems and protocols (19). This study emphasizes the critical importance of investing in the development and readiness of EMTs in the region. Such locally or regionally embedded teams are uniquely positioned to respond to health emergencies more rapidly and effectively, given their shared cultural understanding, language proficiency, and familiarity with the local context. This finding is consistent with the growing body of evidence that underscores the value of regional cooperation and capacity building in public health emergencies. It highlights the need to strengthen regional EMT networks as a sustainable and context-appropriate approach to improving emergency preparedness and resilience (20).

This study systematically mapped the key barriers and enabling factors influencing the EMT classification process, drawing on the participants’ lived experiences and reflections. Through this analytical approach, this study provides an in-depth understanding of the contextual, institutional, and operational determinants that shape the success or delay of EMT classification efforts. The findings offer critical insights that can inform the development of a strategic roadmap aimed at advancing the localization of Emergency Medical Teams (EMTs) and ensuring alignment with the objectives and guiding principles of the EMT 2030 Strategy. Table 2 provides a detailed summary of the identified barriers and enabling factors.

**Table 2:** Challenging and enabling factors for EMT classification process.

Domain	Challenging factors	Enabling factors
Standards	Inflexibility of the classification standards Limited consideration of local realities Variability in interpretation of requirements High resource requirements Difficulty of implementation in conflict or fragile settings Need for specialized expertise and capacities	Regular training initiatives and awareness-raising session Contextualization of standards to fit local realities Development of a “standards explanation bank” to clarify requirements Creation of more opportunities for experience-sharing among teams
Process	Complexity of the classification process Poor communication of procedures and requirements Limited access to technical guidance and support Lack of clarity regarding the strategic value of the classification process Lack of tailored support for applicant organizations Lengthy and costly classification process	Frame the classification as a long-term growth opportunity and a strategic investment in quality Provide support from organizations responsible for classification through a well-defined action plan for applicant organizations Develop structured training programs to build capacity and readiness Raise awareness among decision-makers about the value and implications of classification Offer continuous technical support combined with culturally sensitive mentorship Conduct introductory workshops to orient teams before initiating the classification process
Organization	Internal bureaucracy and administrative delays Financial constraints and limited budget allocation Lack of dedicated human resources for the classification process Competing organizational priorities Inconsistent leadership commitment and follow-up Weak institutional culture of quality improvement Insufficient training and capacity-building opportunities	Timely recruitment of technical experts with relevant field experience Building and retaining a skilled and experienced workforce Drawing on lessons from previous emergency deployments Ensuring internal alignment and coordination across departments Conducting a thorough cost analysis at the initial planning stage Demonstrating strong and visible leadership commitment Establishing robust support services, including WASH and logistics

One prominent barrier identified in this process was the perceived inflexibility of current standards, particularly non-clinical requirements such as full self-sufficiency in WASH, power supply, waste management and logistics. These were designed with high-resource contexts in mind, making them difficult to implement in unstable or conflict-affected LMIC settings. Such operational constraints are well documented in the humanitarian logistics literature, especially in fragile environments where access and infrastructure are unreliable (21). The EMT 2030 Strategy explicitly recognizes the need for context-appropriate adaptation, reinforcing our participants’ call for standards that are technically feasible, culturally relevant, and operationally achievable in low-resource contexts.

The second major theme was the absence of structured training on EMT standards. This represents a fundamental upstream capacity gap. Evidence from emergency preparedness research demonstrates that structured, role-specific training not only improves technical compliance but also strengthens team cohesion and confidence in navigating complex accreditation processes (22, 23) In our study, the lack of training was perceived as a barrier to even initiating the classification journey, and addressing it emerged as one of the most actionable short-term interventions.

Participants also described the classification process as overly complex, with unclear steps, minimal technical guidance, and inconsistent engagement from the assigned mentors. These findings align with accreditation and quality improvement models that emphasize iterative feedback loops and sustained mentor involvement as critical to maintaining progress (24). As a natural consequence, mentorship is not merely technical; it is relational and fosters trust, accountability, and adaptability. The reported absence of detailed, context-specific advice left teams uncertain about whether they were on the right track, contributing to frustration and stalled momentum.

Internal organizational culture is a key determinant of progress. Bureaucratic delays, competing priorities, and shifting leadership focus create bottlenecks that hinder the sustained engagement. Change management literature suggests that without institutional alignment and streamlined decision-making, even well-resourced initiatives struggle to maintain momentum (25). In our study, classification efforts were often sidelined during acute emergencies or donor-driven projects, underscoring the importance of embedding EMT readiness into an organization’s core strategic and operational framework.

Finally, the participants consistently identified greater contextualization as a critical enabler of EMT classification advancement. They emphasized the need for flexible and context-sensitive approaches that reflect local capacities, regulatory frameworks and operational realities. This requires not only adapting the standards themselves but also refining assessment, training, and support mechanisms to align with regional and institutional contexts. Such perspectives echo the broader consensus that rigid, one-size-fits-all models often fail to capture the complex realities on the ground and inadvertently create barriers to progress. Evidence from multiple settings shows that aligning standards and accreditation procedures with an institution’s operational, cultural, and resource contexts enhances their relevance, acceptance, and effectiveness (26). Therefore, fostering context-specific pathways to EMT classification has strong potential to accelerate progress and improve outcomes (27).

Simultaneously, the key enabling factors for the successful implementation of the EMT classification process were identified as the availability of human resources and adequate operational support. These findings align with those reported in the broader accreditation literature, where the presence of skilled personnel and access to necessary resources are consistently cited as critical success factors. The study participants emphasized that without sufficient staffing, particularly individuals with dedicated roles and appropriate technical expertise, the classification process often stalled or lost its momentum. Similarly, the availability of financial and logistical support was viewed as essential for sustaining the necessary preparatory work and ongoing compliance (28).

Another prominent enabler highlighted in this study is the strong and consistent commitment of the leadership. The active engagement of senior leaders not only helps prioritize the classification process within the organization’s strategic agenda, but also fosters a culture of accountability and motivation among the staff. This is in line with the findings of Reisi et al. (29), who noted that leadership commitment plays a pivotal role in driving successful accreditation initiatives by mobilizing resources, setting clear expectations and reinforcing a shared vision. Together, these findings underscore the importance of investing in both human and institutional capacity, while ensuring leadership buy-in, to facilitate a successful and sustainable EMT classification journey.

Our findings also underscore the vital role of close mentorship and peer-to-peer learning in strengthening EMT capacity in LMICs. Structured mentorship, especially when led by experienced practitioners, creates opportunities for real-time guidance, skills transfer, and practical technical guidance, which are particularly valuable in resource-limited and high-pressure environments. Likewise, sharing experiences—both successes and challenges—across teams helps contextualize best practices, avoid repeated mistakes, and adapt strategies more effectively. This approach aligns with lessons learned from previous successful EMT initiatives in LMICs, where mentorship and cross-team collaboration played a critical role in accelerating capacity building, improving response quality, and fostering locally led solutions (30). Our study highlights the importance of embedding the EMT initiative within broader organizational surge mechanisms and positioning it as a core component of institutional strategy. By doing so, organizations can avoid competing priorities, promote coherence across different emergency response efforts, and ensure stronger alignment with existing initiatives. This integrated approach enhances the effectiveness and sustainability of EMTs and reinforces their role in building a more responsive and resilient health system. The findings suggest that treating the EMT classification process as a standalone project limits its potential impact, whereas its full integration into the strategic and operational fabric of an organization could set the foundation for a more coordinated and agile emergency response (31). Our study reflects many of the lessons observed in the broader development of EMTs over the past decade. We found that establishing and operationalizing EMTs is not a one-size-fits-all process; it is shaped by both global best practices and specific realities on the ground. One of the main findings was the importance of tailoring approaches to local contexts, involving a wide range of stakeholders, and committing to long-term training and preparedness efforts. Similar to what has been observed in other regions, our research points to governance, workforce capacity, sustainable funding, and coordination between actors as key elements in building effective emergency-response systems. These findings highlight the importance of continued flexibility, consistent investment in national capacities, and strong collaboration at the regional and international levels. As the EMT initiative grows and evolves, maintaining a focus on learning, adaptability, and integration into existing health systems will be essential for their continued impact and sustainability (32).

## 5 Conclusion

The EMT initiative serves as a vital surge mechanism to support timely and effective medical responses during emergencies. Despite its recognized importance, the MENA region remains behind in progress, with only one team currently classified according to global EMT standards. Accelerating progress requires a strategic and integrated approach that embeds EMTs within national surge systems and contextualizes global standards to meet local realities. Strengthening leadership, logistics, and human resources, alongside mentorship and peer learning, will be key to building sustainable capacity. By advancing these areas, the region can better integrate EMTs into its emergency response architecture, enhance operational readiness, and contribute to improved regional and global health security.

### 5.1 Limitation

This qualitative study is subject to the inherent limitations of purposeful sampling and semi-structured interviews. Purposeful sampling introduces selection bias and limits generalizability, whereas semi-structured interviews are vulnerable to interviewer and social desirability biases. The subjective nature of qualitative data analysis and the small sample size further restricts the transferability of the findings to other contexts. These limitations were mitigated through reflexivity, member checking, and transparent documentation of research methods.

## Data Availability

The datasets presented in this study can be found in online repositories. The names of the repository/repositories and accession number(s) can be found in the article/Supplementary material.
