# Supplementary material for: Advancing emergency medical team classification in MENA region: a qualitative study
Source: Front Public Health. 2026 May 19;14:1820087. doi: 10.3389/fpubh.2026.1820087 (PMC13270535; doi:10.3389/fpubh.2026.1820087)
Supplement: Supplementary file 1 [file Supplementary_file_1.docx]

Supplementary Material

1. Interview guide
2. Staff working in the EMT formation project
3. Management level staff
4. Technical sectors involved in EMT (logistics, medical, admin, etc.)

Objective

1. Understand the challenges with EMT standards in MENA
2. Map the obstacles of hard to obtain standards

Initiation of the interview

1. Presentations round
2. Presentation of the study aim and the objective of the interview
3. Request for permission to audio-record the session (with clarification on anonymity and confidentiality)
4. Sign the consent form

Questions

*Demographic*

1. Professional & educational background
2. Position at organization
3. Years of experience in the humanitarian field
4. What is your role in the EMT project?
   1. Which part of the standardization process are you involved in?

Task and Competencies in EMT Standardization

1. How important is it to have standardized EMTs?
   1. What are the main motives for your organization to meet these standards?
2. What are your perceptions of the standardization process? (complicated, lengthy, easy, necessary, etc.)
3. Did you receive any training on the EMT standardization process?
   1. What was the training about?
4. If you have a written plan of action for the EMT standardization process, what are the components of this plan?
   1. Elaborate on the components of the plan of action
   2. How do you implement the plan in practice?

*Standard details*

1. *Can you describe your experience working towards complying with EMT standards?*
   1. *What factors facilitate meeting the standards? (e.g., management support, external technical support, availability of funds, etc.)*
      1. *How do these factors contribute to meeting the standards?*
   2. *What factors make it challenging to meet the standards?*
      1. *How do these factors challenge the process of meeting the standards?*
2. *Which standard/s are considered the most challenging to meet? Why?*

- *For each of the named standards, the following were explored:*

1. *Hard to understand*
2. *Resources intense*
3. *Technical guidance availability*
4. *other reasons...*
5. Do you see any of the standards as inappropriate or irrelevant within your context? Why?
6. What are the internal challenges within your organization that make it difficult to meet the standards?
7. Human resources, technical capacity
8. Support services (logistics, admin)
9. Resources, funds
10. Do you believe that developing SOP contributes to the standardization process? Why?

*Recommendation*

1. What organizational capacities do you believe are essential to meet EMT standards?
2. According to your experience, how can resources be better managed in the process of standardization?
3. According to your experience, what is the best way to prepare an organization for EMT accreditation?
4. What are the next steps for your organization to proceed with EMT standardization?
5. **Consent form**

• I……………………………………… voluntarily agree to participate in this research study.

• I understand that even if I agree to participate now, I can withdraw at any time or refuse to answer any question without any consequences.

• I understand that I can withdraw permission to use data from my interview any time before publication of research findings , in which case the material will be deleted.

• I have had the purpose and nature of the study explained to me and I have had the opportunity to ask questions about the study.

• I understand that I will not benefit directly from participating in this research

. • I agree to my interview being audio-recorded.

• I understand that all information I provide for this study will be treated confidentially.

• I understand that in any report on the results of this research my identity will remain anonymous.

• I understand that disguised extracts from my interview may be quoted in dissertation, conference presentation, published papers

• I understand that signed consent forms and original audio recordings will be retained in CREMIDEM research centre until paper publication and PhD dissertation

• I understand that under freedom of information legalisation I am entitled to access the information I have provided at any time while it is in storage as specified above.

• I understand that I am free to contact the main researcher involved in the research to seek further clarification and information.

Main researcher. Dr.Mohamed Abdelaziz, CRIMEDIM , [Rashadmohamd@gmail.com](mailto:Rashadmohamd@gmail.com), phone number :+201067235588

Signature of research participant

----------------------------------------- ---------------- Date

Signature of researcher I believe the participant is giving informed consent to participate in this study ------------------------------------------ ---------------------- Date
